# Dietary restriction induces persistent multigenerational phenotypic effects in *Phytoseiulus persimilis*

**DOI:** 10.1002/ps.70923

**Published:** 2026-05-17

**Authors:** Xia Chen, Keshi Zhang, Xiao Han, Yun Xu, Zhi‐Qiang Zhang

**Affiliations:** ^1^ Institute of Plant Protection Fujian Academy of Agricultural Sciences Fuzhou China; ^2^ Manaaki Whenua – Landcare Research Group Bioeconomy Science Institute Maiangi Taiao Auckland New Zealand; ^3^ School of Biological Sciences University of Auckland Auckland New Zealand; ^4^ College of Plant Protection Jilin Agricultural University Changchun China; ^5^ Institute of Forest Protection Fujian Academy of Forestry Sciences Fuzhou China

**Keywords:** dietary restriction, life‐history traits, maternal effects, mating history, paternal effects, *Phytoseiulus persimilis*

## Abstract

**BACKGROUND:**

Transgenerational phenotypic plasticity (TGP) allows environmental effects to persist across generations, yet the extent of diet‐induced TGP remains incompletely resolved. Prey availability is a key determinant of predator performance in both mass‐rearing and field environments, potentially influencing the reliability of pest suppression. Here, we examined how ancestral dietary restriction affects life‐history traits in the predatory mite *Phytoseiulus persimilis*, a widely used biological control agent of the two‐spotted spider mite *Tetranychus urticae*.

**RESULTS:**

Founding (F_0_) and first‐generation (F_1_) individuals were reared under low‐ or high‐prey diets, followed by three generations (F_2_–F_4_) under abundant prey to simulate recovery. In F_4_, individuals from high‐prey ancestral lineages exhibited higher survival and larger body size, although size effects were restricted to females. In a second experiment, F_5_ offspring from crosses manipulating maternal and paternal ancestral diet histories and paternal mating history showed that maternal diet consistently influenced egg size, female developmental duration, and size at maturity. Paternal effects were context‐dependent, interacting with maternal lineage and mating history to affect offspring traits.

**CONCLUSION:**

Poor ancestral diet can exert persistent, sex‐specific effects on predator performance even after multiple generations of favorable conditions, with maternal influences generally stronger than paternal effects. These findings highlight the complexity of TGP and the importance of considering multigenerational dietary legacies in mass‐rearing and field deployment of predatory mites. Optimizing diet during rearing and accounting for ancestral nutritional history may enhance the consistency and effectiveness of biological control programs. © 2026 The Author(s). *Pest Management Science* published by John Wiley & Sons Ltd on behalf of Society of Chemical Industry.

## Graphical abstract

The study shows ancestral dietary restriction in *Phytoseiulus persimilis* produces persistent, sex‐specific, multi‐generational effects on survival and body size, with stronger maternal than paternal lineage influences despite nutritional recovery.

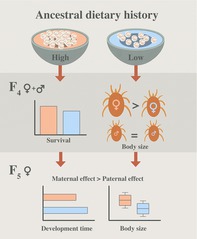

## 1 INTRODUCTION

The effectiveness of biological control agents depends on their ability to maintain consistent performance under variable environmental conditions.^1^, ^2^, ^3^ Natural enemies frequently experience fluctuations in prey availability and diet quality during both mass rearing and field deployment.^4^, ^5^ Such variability can influence predator development, survival, and reproductive output, ultimately determining the success of pest suppression.^6^, ^7^, ^8^, ^9^ Importantly, these environmental effects may extend beyond a single generation.^10^, ^11^ When parental environmental conditions influence offspring traits without changes in DNA sequence, this process, known as transgenerational phenotypic plasticity (TGP), can affect the long‐term efficacy.^12^, ^13^, ^14^, ^15^

Among the mechanisms underlying TGP, condition transfer, the transmission of parental condition to offspring, is particularly relevant to pest management.^16^, ^17^, ^18^, ^19^ Parental condition can be shaped by intrinsic factors such as age and health, as well as environmental factors including diet and stress.^16^, ^17^, ^18^, ^19^ High‐condition parents often produce larger or better‐provisioned offspring with improved survival and performance, whereas low‐condition parents may transmit constraints that persist across generations.^17^, ^20^, ^21^, ^22^ Specifically, offspring quality can influence population growth of predators and the strength of pest suppression.^1^

Diet is one of the most influential environmental factors shaping both within‐generation performance and transgenerational responses.^23^, ^24^, ^25^, ^26^ In predatory arthropods, diet quality and prey availability strongly affect key life‐history traits, including developmental duration, fecundity, and survival, all of which are critical for effective pest management.^4^, ^7^ Moreover, diet‐induced effects may persist across generations, influencing predator population dynamics.^10^, ^21^, ^27^, ^28^ For biological control systems, this has important implications, as suboptimal diets during mass rearing or temporary prey limitation in the field may have adverse multigenerational effects for predator performance.^10^, ^11^ However, the persistence, reversibility, and predictability of diet‐induced TGP remain unresolved.^13^, ^29^, ^30^

Although maternal effects have been more extensively studied, paternal effects are increasingly recognized as important contributors to offspring traits.^17^, ^31^, ^32^, ^33^, ^34^, ^35^, ^36^, ^37^ Paternal condition, shaped by diet, age, or mating history, has been shown to influence offspring fitness, physiology, and metabolism across a wide range of taxa.^34^, ^38^, ^39^, ^40^, ^41^, ^42^ In biological control programs, where mating structure and male condition may vary during mass production, these paternal effects may influence both rearing efficiency and field performance, yet they remain insufficiently explored.

The predatory mite *Phytoseiulus persimilis* Athias‐Henriot (Acari: Phytoseiidae) is widely used for the control of the two‐spotted spider mite, *Tetranychus urticae* Koch (Acari: Tetranychidae).^43^, ^44^ Its short generation time and sensitivity to prey availability make it a suitable model for examining how parental and ancestral diet influence offspring traits relevant to pest suppression, including developmental duration and size at maturity.^5^, ^6^, ^11^

This study adopts a multigenerational approach to address key gaps in understanding diet‐induced TGP in biological control systems. In the first experiment, we investigated whether diet‐induced transgenerational effects persist or dissipate after several generations of recovery under favorable dietary conditions, simulating scenarios in which predators experience temporary prey limitation followed by resource abundance in cropping systems. We hypothesized that differences between high‐ and low‐condition lineages would diminish after multiple generations of recovery (Hypothesis 1).

In a second experiment, we further examined whether maternal and paternal lineages differentially mediate diet‐induced transgenerational effects and whether variation in male mating history influences female reproductive output and offspring development. These factors are directly relevant to optimizing mass‐rearing protocols and ensuring consistent predator quality. We hypothesized that (i) the persistence of ancestral dietary effects would differ between maternal and paternal lineages (Hypothesis 2), and (ii) increased male mating frequency would not significantly influence female reproductive performance or performance of their immediate offspring (Hypothesis 3), consistent with previous findings.^45^

By integrating diet, parental effects, and multigenerational responses, this study provides insight into how environmental history shapes predator performance across generations, with direct implications for improving the reliability and effectiveness of biological control strategies.

## 2 MATERIALS AND METHODS

### 2.1 Study species and rearing conditions

We used a laboratory‐maintained population of *P. persimilis* that had been in culture for approximately a year before the experiment (>30 generations). Both *P. persimilis* and its prey, the two‐spotted spider mite, were originally obtained from mass‐reared populations (Bioforce Limited, Karaka, Auckland). Colonies of *T. urticae* were maintained on potted bean plants (*Phaseolus vulgaris* L.), while *P. persimilis* colonies were reared on detached bean leaves heavily infested with *T. urticae*. These leaves were placed on a black plastic sheet (approximately 15 cm × 10 cm), supported by a water‐saturated sponge within a plastic tray (22 cm × 22 cm × 3.5 cm) filled with water to prevent mite escape. All colonies were maintained at 20°C ± 2°C, 65–75% relative humidity (RH), and a 12:12 h light–dark photoperiod.

### 2.2 Establishment and maintenance of dietary lineages

**Figure 1:**
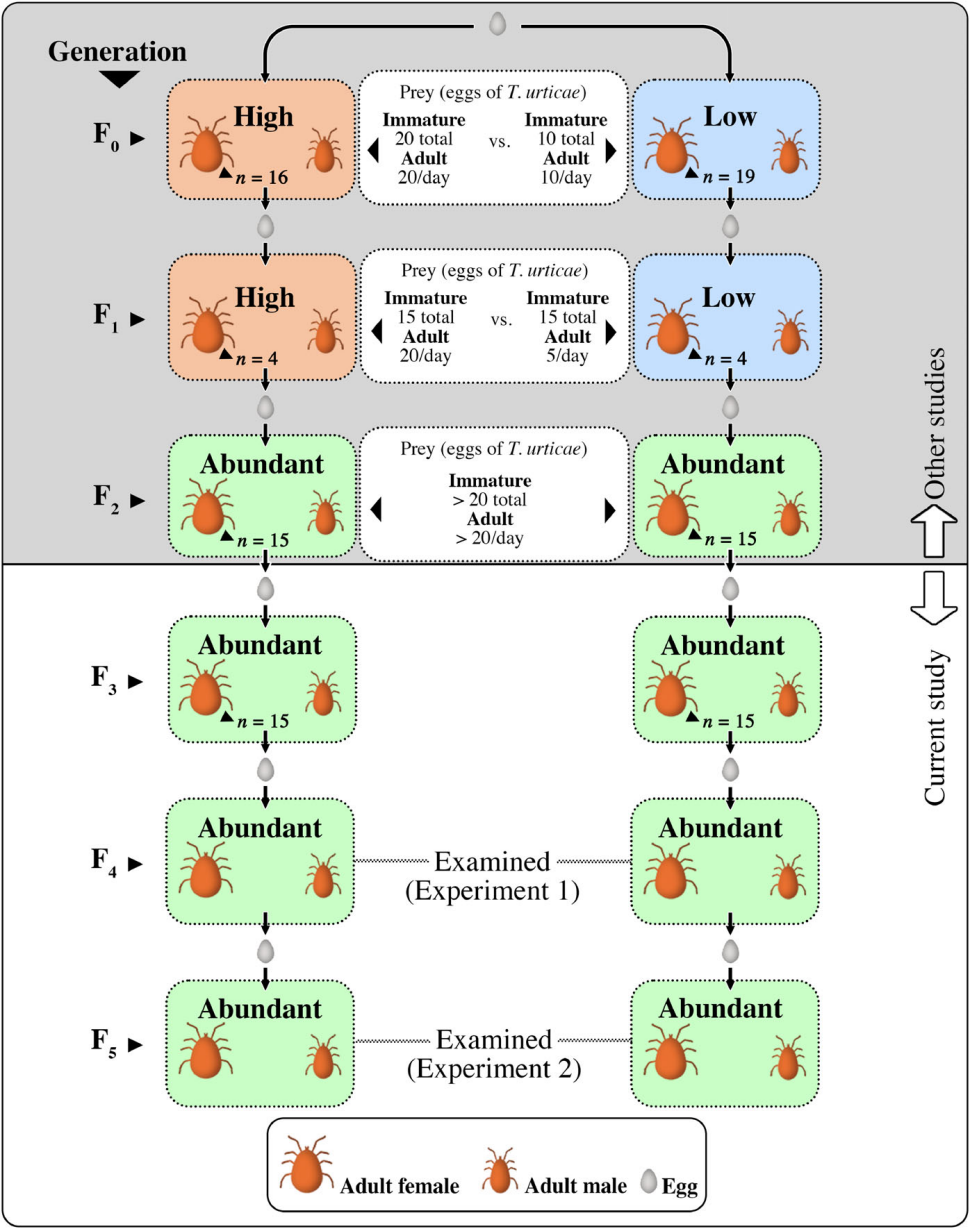
A schematic representation of the transgenerational dietary manipulation across six generations. Founding individuals (F_0_) were exposed to either a low‐ or high‐quality diet, and this diet was maintained through F_1_. Subsequent generations F_2_–F_5_ were reared on a standard diet with equal prey abundance, eliminating further dietary differences. F_4_ (Experiment 1) and F_5_ (Experiment 2) generations were assessed to determine whether the influence of ancestral dietary restriction had dissipated.

Two ancestral dietary conditions, High and Low, were given to F_0_ and F_1_ generations (Fig. 1).^6^, ^11^ From F_2_ to F_5_, both lineages were maintained under abundant dietary conditions (i.e., a total of >20 *T. urticae* eggs provided throughout development, and > 20 eggs provided daily during adulthood). All individuals were reared from egg to adult in custom rearing cells (Fig. S1(a)).^8^

Upon reaching adulthood, virgin males and females (<24 h post‐emergence) were paired in new rearing cells for 24 h with a mate from the same dietary lineage. During mating, abundant prey (>30 eggs) was provided. After mating, each female was transferred individually to a custom rearing tube (Fig. S1(b)) for oviposition.^6^, ^46^ Eggs (<24 h old) were collected and used to initiate the next generation. Females were allowed to oviposit for 1 week, and a random subset of eggs was selected to continue the lineage. All experimental units were maintained in sealed containers with saturated salt solutions to ensure approximately 80% RH, and incubated at 25 ± 1°C with a 12:12 h light–dark cycle.^47^

### 2.3 Experiment 1: effects of ancestral diet on F_4_ traits

To examine carry‐over effects of ancestral dietary history, we reared 95 eggs from the High‐diet lineage and 102 eggs from the Low‐diet lineage, laid by F_3_, under abundant prey conditions (>20 *T. urticae* eggs) until adulthood. Survival to maturity, sex ratio, and body size (measured as dorsal plate length) were recorded. Each cell was checked twice daily (9:00 and 16:00) to monitor development. Although mating procedures were part of Experiment 2, size measurements of F_4_ adults, taken after mating, were included in the analyses for Experiment 1. Adults were slide‐mounted in Hoyer's medium and oven‐dried at 40°C for 1 week.^48^ Dorsal plate length was measured under a phase‐contrast microscope (Eclipse 90i, Nikon Corporation, Japan) at 200× magnification using NIS‐Elements software (v5.10).

### 2.4 Experiment 2: effects of sex‐specific ancestral diet and paternal mating history on F_4_ reproductive traits and F_5_ developmental traits

**Figure 2:**
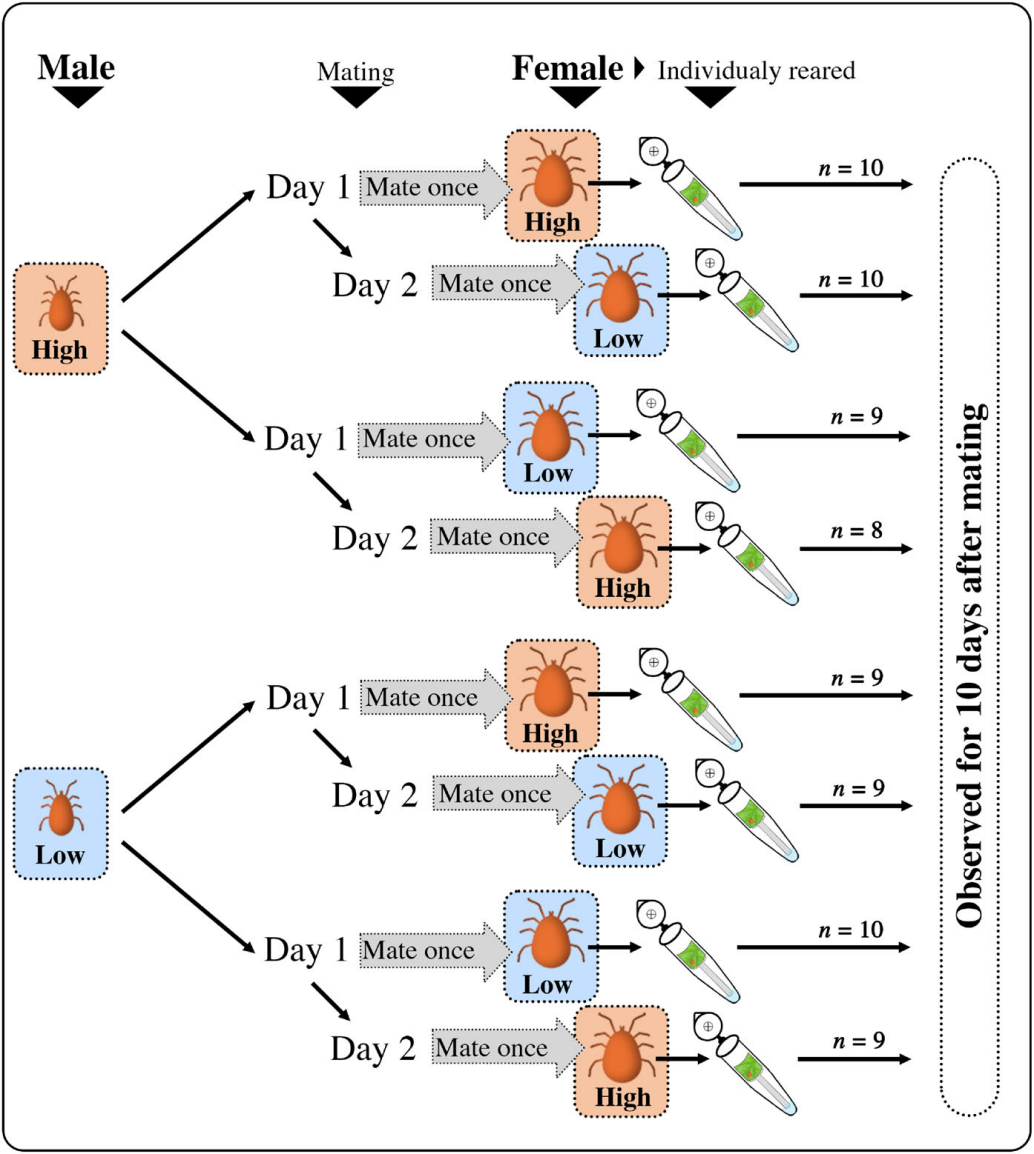
A schematic representation of the study design for pairing F_4_ generation. Individuals were produced using reciprocal crosses to disentangle maternal and paternal lineage effects. Males were mated with two females (one for each day of Day 1 and Day 2) with alternate dietary lineage (Low–low quality diet; High–high quality diet). The number of successfully mated and ovipositing females for each treatment is given (*n*). Refer to the text for a detailed description of the dietary history.

To disentangle maternal and paternal influences, virgin adults (<24 h old) from the F_4_ generation were paired either within or between ancestral dietary histories (Fig. 2). Each pair was mated for 24 h in new rearing cells. On the second day, males were paired with a second virgin female from the *alternate* dietary lineage to assess the influence of paternal mating history on offspring outcomes.

After mating, males were slide‐mounted for size measurement using the same protocol as in Experiment 1. Females were individually housed in rearing tubes and allowed to oviposit for 10 days, after which they were also slide‐mounted for size measurement. For F_4_ females, we measured three reproductive traits following mating: pre‐oviposition period (time from mating to first egg), daily oviposition rate (mean eggs/day over the 10‐day period), and maximum daily oviposition (peak egg output in a single day).

Eggs (the F_5_ generation) were collected daily. A subset of 30 eggs per treatment was randomly selected for size measurement (length [*L*] and maximum breadth [*B*]) under 200× magnification. Egg volume (*V*) was estimated using the following formula^49^:

$$V=\left(0.6057–0.0018B\right){\mathrm{LB}}^{2}$$

Additionally, 12 eggs per treatment were randomly selected and individually reared with abundant food to adulthood. Developmental duration (time from egg to adult, in days) and size at maturity were recorded for female offspring only, due to a female‐biased sex ratio (>60–100% females) and small sample size for males. The potential non‐independence of offspring originating from the same parental pair or female was not accounted for in the analyses.

### 2.5 Statistical analysis

All analyses were conducted in R using RStudio (v2023.12.1).^50^ Figures were produced using the *ggplot2* package.^51^ In Experiment 1, binary traits (immature survival and sex ratio) were analyzed using logistic regression. A two‐way ANOVA (ancestral diet × sex) assessed the effects on F_4_ size at maturity, with *post hoc* comparisons via Tukey's honestly significant difference (HSD) test. In Experiment 2, three‐way aligned rank transform (ART) ANOVAs (using the *ARTool* package)^52^ were applied to F_4_ female traits (pre‐oviposition period, daily oviposition, maximum oviposition), with fixed factors: female ancestral diet, male ancestral diet, and male mating history. Nonparametric ART ANOVA was used due to small sample sizes and non‐normal distributions. For F_5_ traits (egg volume, developmental duration, and size at maturity), three‐way ANOVAs were used with fixed effects: maternal diet, paternal diet, and paternal mating history. The developmental durations were log‐transformed to meet model assumptions. An alpha level of 0.05 was used for all significance testing.

## 3 RESULTS

### 3.1 Experiment 1: effects of ancestral diet on F_4_ traits

F_4_ offspring descended from the ‘High’ ancestral diet treatment exhibited significantly higher survival to adulthood than those from the ‘Low’ treatment (logistic regression: Wald *χ* ^2^ = 3.984, *df* = 1, *P* = 0.046; Table 1). However, sex ratio was not significantly influenced by ancestral dietary history (Wald *χ* ^2^ = 0.352, *df* = 1, *P* = 0.553).

**Table 1:** Survival to adulthood and sex ratio of *Phytoseiulus persimilis* F_4_ generation derived from different ancestral (F_0_–F_1_) dietary treatments (high quantity–High; low quantity–Low)

Dietary history	*n*	Immature survival (%)	Proportion of females (%)
High	95	87.37	75.90
Low	102	76.47	71.79

**Figure 3:**
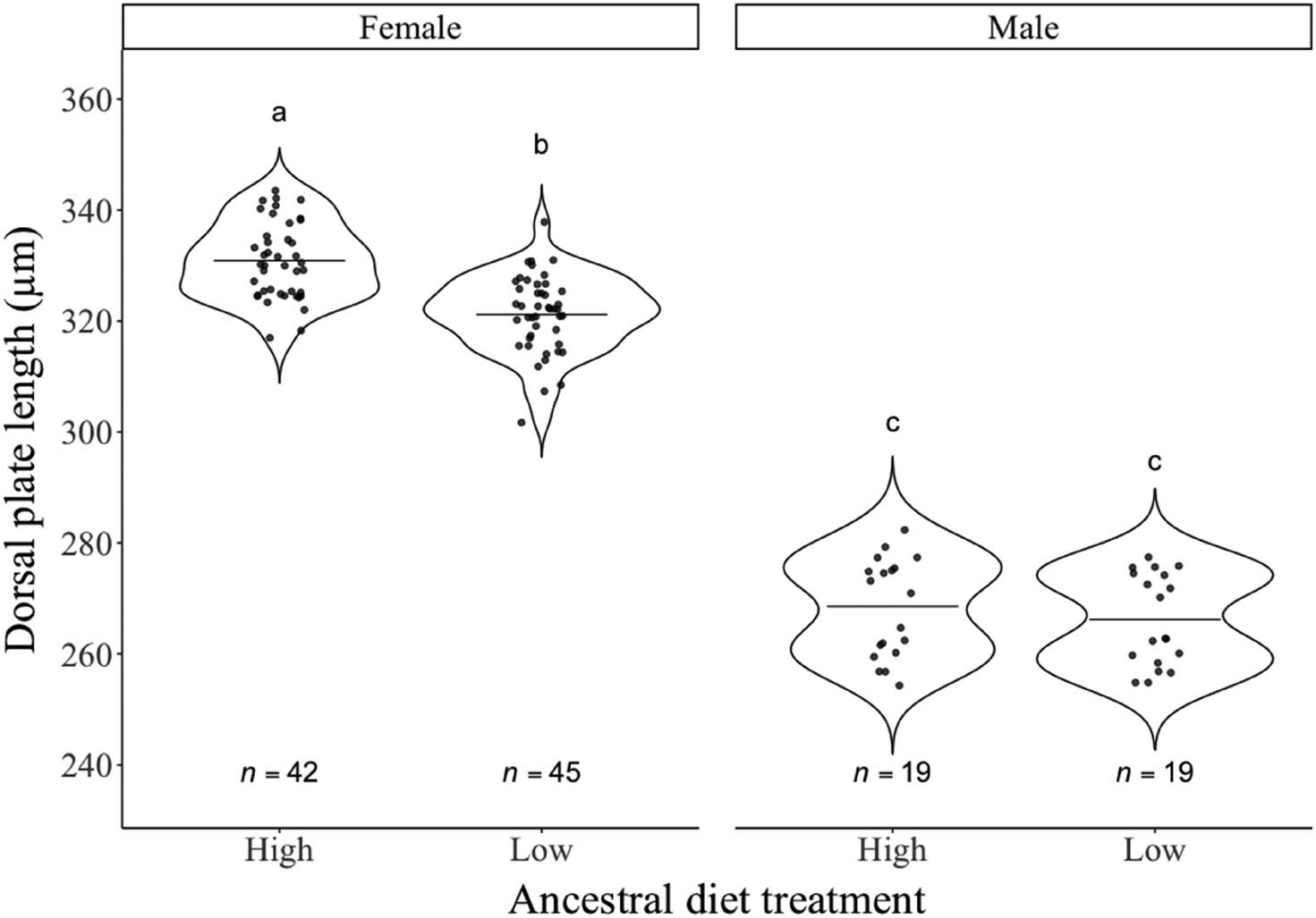
Effect of ancestral diet on F_4_ body size at maturity (dorsal plate length [μm]). High and Low ancestral dietary treatments (for F_0_–F_1_). Each violin shows the density of observations; dots represent individual sizes. Horizontal lines represent group means. Letters denote statistically significant differences based on Tukey's HSD *post hoc* test (*P* < 0.001).

Body size (as estimated by dorsal plate length) differed significantly between the sexes, with females being larger than males (ANOVA: *F*_1_,_121_ = 1684.739, *P* < 0.001). Individuals from the High ancestral diet treatment were also significantly larger than those from the Low treatment (*F*_1_,_121_ = 25.466, *P* < 0.001). There was a significant interaction between sex and ancestral diet (*F*_1_,_121_ = 6.634, *P* = 0.011), such that the effect of ancestral diet on size was only evident in females (Fig. 3).

### 3.2 Experiment 2: effects of sex‐specific ancestral dietary history and paternal mating history on F_4_ reproductive traits and F_5_ offspring traits

Neither the female's own ancestral dietary history, the partner's ancestral dietary history, nor the partner's mating history had significant main effects on pre‐oviposition period, daily oviposition rate, or maximum daily oviposition (Tables S1 and S2). A significant interaction was detected between male's ancestral dietary history and male's mating history (Table S2), but pairwise comparisons revealed no statistically significant differences among treatment groups.

**Figure 4:**
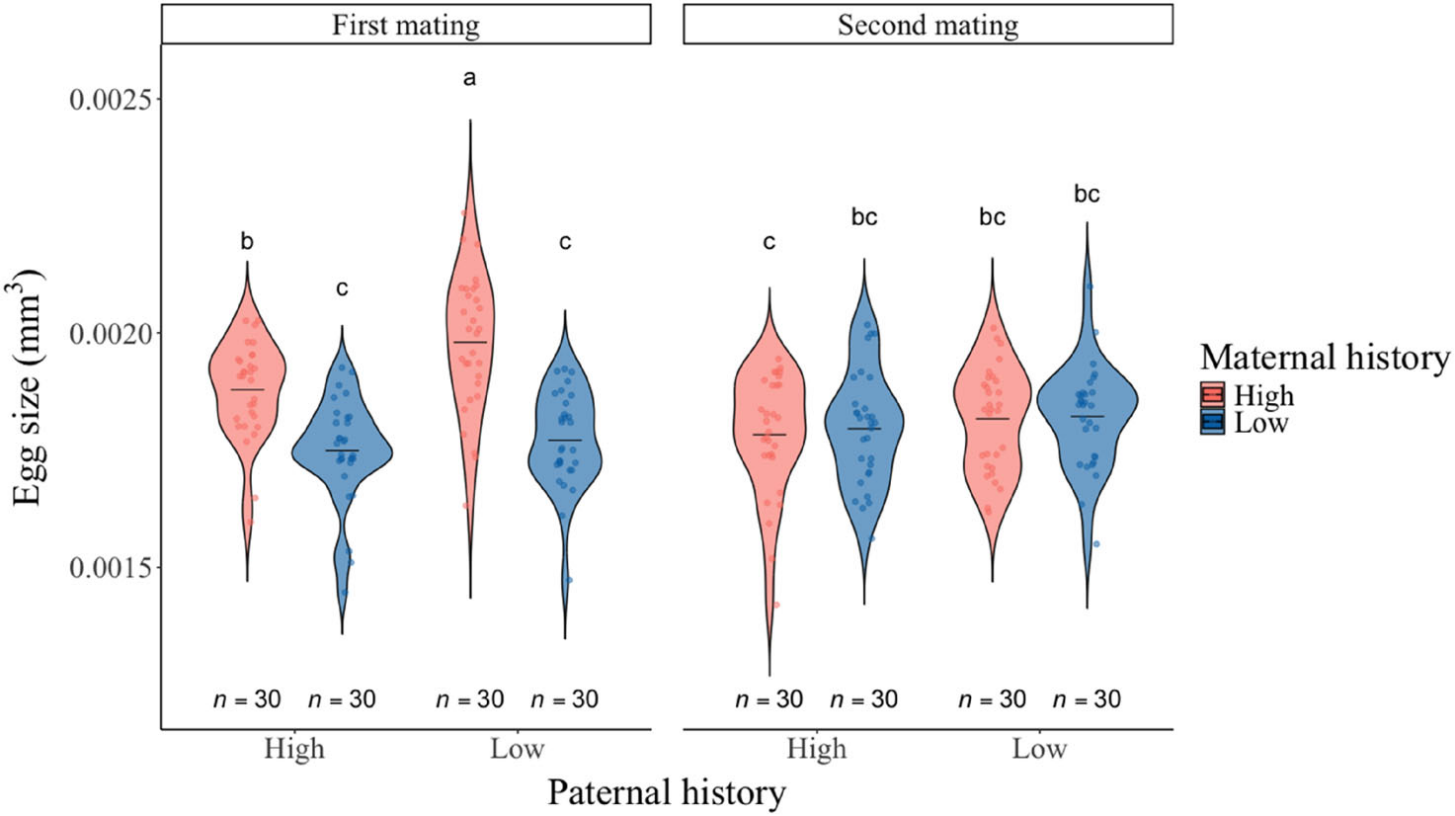
Egg size (mm^3^) produced by F_4_ females as influenced by maternal‐line and paternal‐line dietary history (High or Low) and paternal mating history (first *vs*. second mating). Each violin shows the density of observations; dots represent individual eggs. Horizontal lines represent group means. Letters denote statistically significant differences based on Tukey's HSD *post hoc* test (*P* < 0.05).

Egg size was significantly influenced by maternal‐line dietary history, paternal‐line dietary history, and paternal mating history (Table S3). Females with a High ancestral dietary history and those mated with virgin males (first mating) produced larger eggs than females with a Low dietary history or those mated with previously mated males (second mating). In contrast, High paternal dietary history was associated with smaller egg size (Fig. 4). A significant interaction between maternal‐line dietary history and mating history was detected (*F*_1_,_232_ = 34.986, *P* < 0.001), where maternal effects were only apparent when females mated with virgin males (Fig. 4).

**Figure 5:**
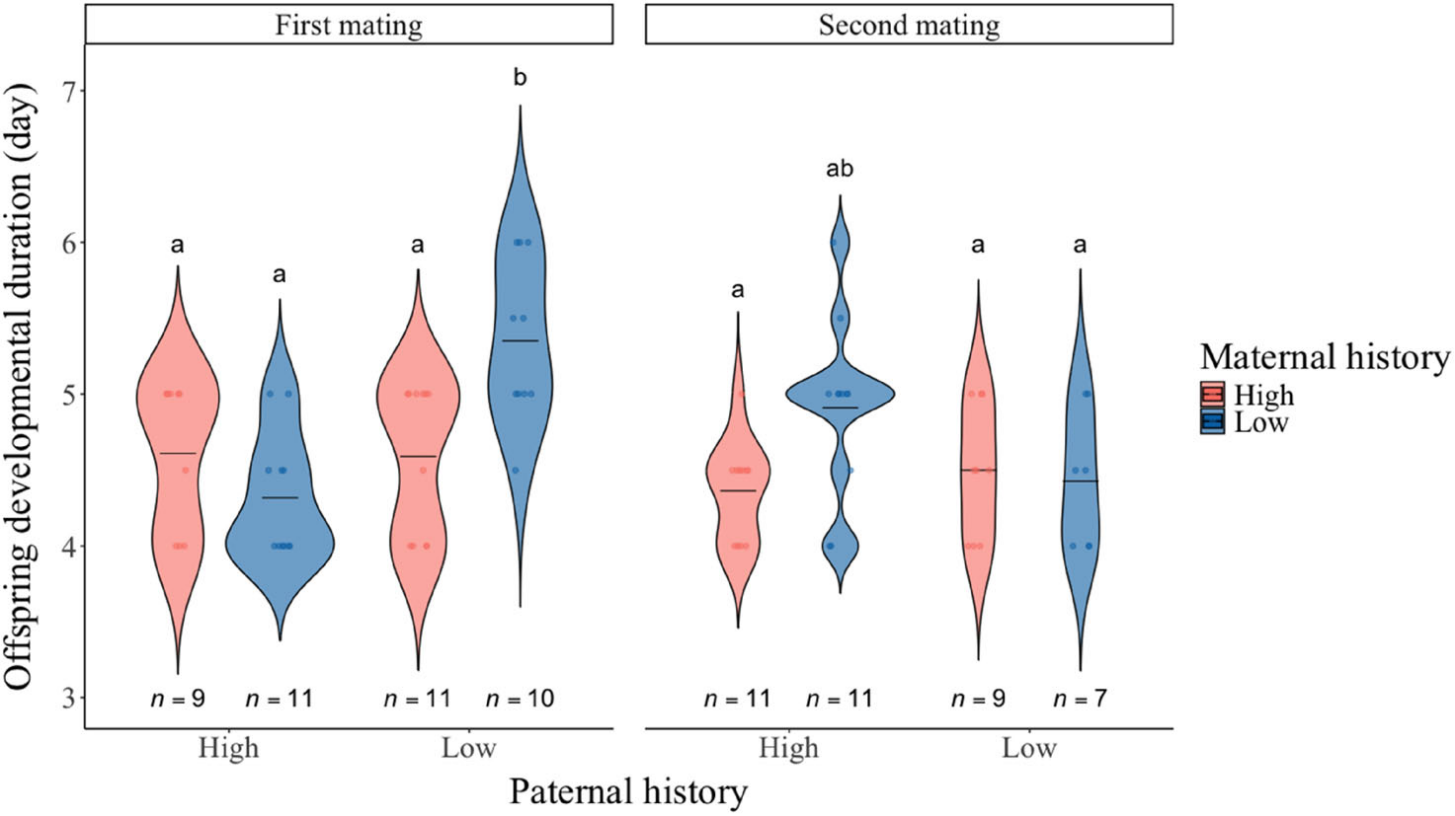
Developmental duration (day) of F_5_ females as influenced by maternal‐line and paternal‐line dietary history (High or Low) and paternal mating history (first *vs*. second mating). Each violin shows the density of observations; dots represent individual eggs. Horizontal lines represent group means. Letters denote statistically significant differences based on Tukey's HSD *post hoc* test (*P* < 0.05).

Maternal‐line dietary history was the only significant main effect on developmental duration in F_5_ females (Table S4), with daughters of Low‐history mothers taking longer to reach maturity (Fig. 5). Paternal effects were dependent on both mating history and maternal‐line dietary history, with significant two‐way and three‐way interactions detected. A prolonged developmental period was only evident when males had Low dietary ancestry and mated as virgins, particularly when maternal history was also Low (Fig. 5).

**Figure 6:**
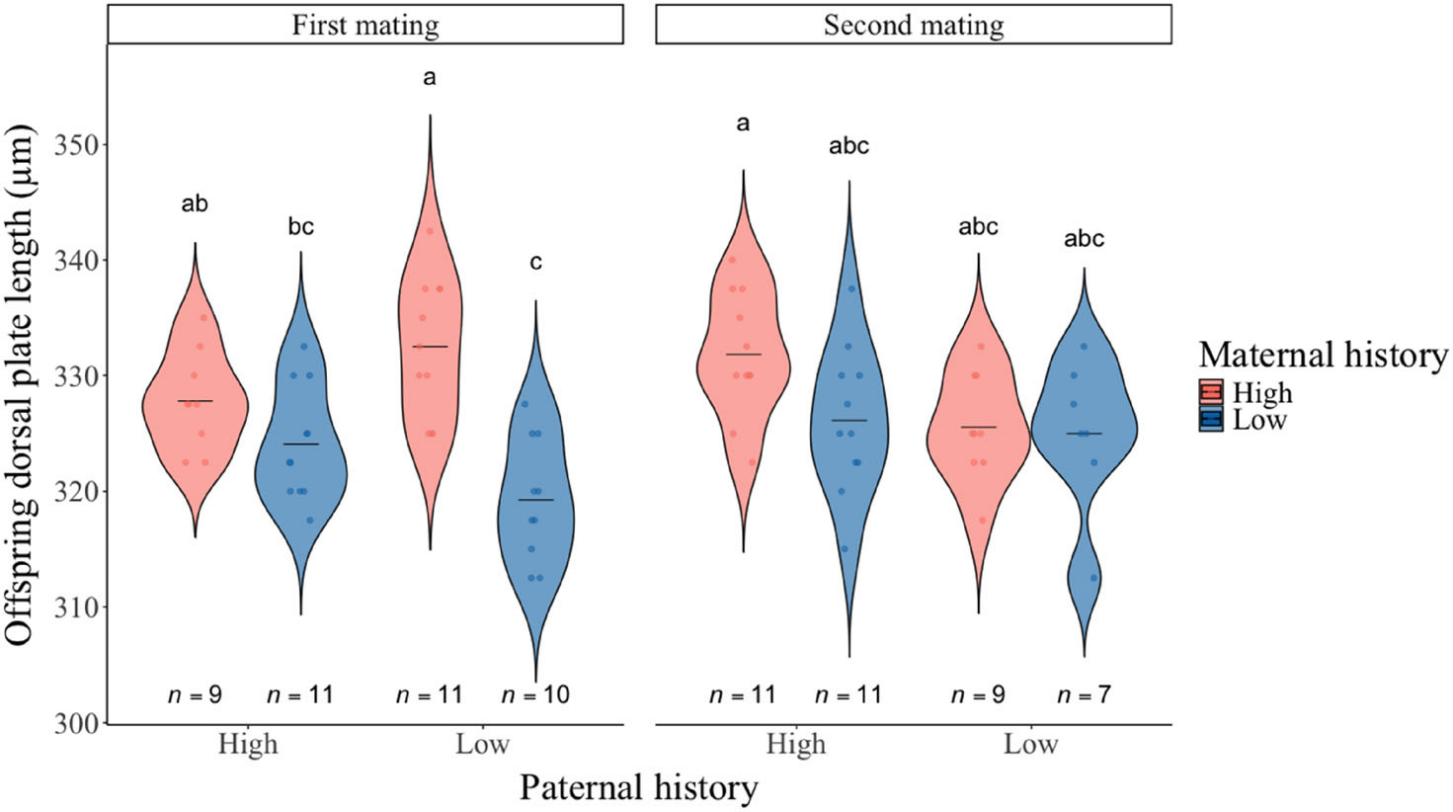
Size at maturity (dorsal plate length [μm]) of F_5_ females as influenced by maternal‐line and paternal‐line dietary history (High or Low) and paternal mating history (first *vs*. second mating). Each violin shows the density of observations; dots represent individual eggs. Horizontal lines represent group means. Letters denote statistically significant differences based on Tukey's HSD *post hoc* test (*P* < 0.05).

Similarly, female size at maturity in the F_5_ generation was significantly influenced **only** by maternal‐line dietary history (Table S5), with daughters from High‐history mothers being larger (Fig. 6). A significant three‐way interaction was detected, suggesting that paternal effects depended on both mating history and maternal‐line dietary history. Paternal influence was most evident during the first mating and varied depending on the mother's ancestral dietary background (Fig. 6).

## 4 DISCUSSION

Dietary history influenced predator performance beyond the immediate generation, with individuals from the High lineage showing higher survival and larger body size than those from the Low lineage, even after several generations under favorable conditions. However, partial mortality in the Low lineage during earlier generations may have introduced unintended selection, which may have contributed to the observed divergence between lineages. Notably, this size difference was restricted to females, indicating a sex‐specific response. From a biological control perspective, these results suggest that nutritional conditions experienced during earlier generations may influence the quality of predators used in pest suppression programs. Larger female body size is often associated with higher fecundity, improved prey consumption, and enhanced population growth, all of which are critical for effective control of pest populations.^53^, ^54^, ^55^, ^56^ Therefore, the persistence of such differences may have practical implications for both mass‐rearing systems and field performance, particularly if suboptimal diets reduce the long‐term quality of released individuals.

The transgenerational influence on female body size observed in this study may reflect a sex‐specific effect or greater plasticity in female size relative to males. In *P. persimilis*, sex‐specific maternal effects have been documented, with food‐restricted mothers producing smaller eggs for daughters but not sons.^57^ Moreover, females are naturally larger than males, and female size is more plastic in response to prey availability.^5^, ^58^ In contrast, male size variation is more restricted, possibly due to sexual selection pressures that disadvantage smaller males. Mechanisms underlying such dimorphism may involve differential parental provisioning, sex‐specific plasticity thresholds, or selection favoring sex‐specific traits.^54^, ^58^, ^59^ Given that female size influences mating behavior in *P. persimilis*,^54^ the persistence and fitness consequences of this sex‐specific influence merit further investigation.

When maternal and paternal lineages were examined separately, maternal dietary history had a stronger and more consistent influence on offspring traits, particularly developmental duration, size at maturity, and egg size. This is consistent with the expectation that maternal provisioning plays a central role in shaping offspring quality.^60^, ^61^, ^62^ Although paternal effects were less pronounced, they were not negligible. Paternal lineage influenced egg size, and interactions between maternal and paternal history suggest that offspring traits are shaped by a combination of parental conditions.^63^, ^64^

Male mating history and ancestral diet had limited direct effects on short‐term fecundity, but interactions with mating order suggested that dietary history may influence male fertility across successive copulations. Reduced fertility in later matings could reflect depletion of sperm reserves or slower replenishment under nutritional stress, as observed in other arthropods.^65^, ^66^, ^67^ However, this remains speculative, as sperm traits and fertilization success were not directly measured in this study. In mass‐rearing environments, where males may mate multiple times, such effects could reduce fertilization success and lead to variability in offspring production. Given that *P. persimilis* exhibits moderate polygyny,^68^ ensuring adequate male nutrition may be important not only for maintaining mating activity but also for sustaining fertility across repeated matings.

The persistence of dietary effects across generations suggests that predator performance may be influenced by prior rearing conditions, even after diet improves. For biological control programs, this indicates that temporary nutritional stress, such as during colony establishment or fluctuations in prey supply, may have delayed consequences for predator quality. This has practical implications for the design of rearing protocols, particularly the need to maintain consistent and high‐quantity diets across generations to avoid unintended carry‐over effects.

Several limitations should be acknowledged. The conclusions are based on indirect evidence, and further work is required to clarify the underlying physiological mechanisms. Further studies using split‐brood designs in *P. persimilis* and other sexually reproducing species could help account for potential genotype‐related variation.^69^ The dietary treatments applied to early generations were not fully standardized across life stages, which may have introduced confounding effects. Measurements focused primarily on female traits, limiting insight into male performance and fertility. Finally, the relatively short observation period may underestimate longer‐term effects on traits such as lifetime fecundity, longevity, and stress tolerance, which are also relevant for biological control.

Future studies should extend observations across the full lifespan and incorporate direct measures of reproductive performance, including male fertility and mating dynamics. Investigating how different levels and durations of dietary restriction influence predator quality will also help define thresholds relevant to mass‐rearing and field conditions. Understanding how nutritional history shapes predator performance will be essential for improving the consistency and effectiveness of biological control programs.

Overall, these findings demonstrate that dietary conditions experienced across generations can influence key life‐history traits in *P. persimilis*, with maternal effects playing a dominant role and paternal contributions adding further complexity. From an applied perspective, maintaining optimal nutrition during rearing and considering parental condition may be critical for ensuring predator quality and reliable pest suppression outcomes.

## Supporting information


**Figure S1.** Experimental rearing arenas for *Phytoseiulus persimilis*. (a) Rearing cells used for immature development and adult mating (1♀ + 1♂). Each cell comprised a plexiglass slide (25 × 20 × 1 mm) with a central cavity (6 mm diameter) covered by a smaller glass slide (20 × 15 × 1 mm). A black cotton cloth was affixed to the base for contrast and ventilation. Two metal clips held the slides together. (b) Rearing tubes used for individual adult maintenance. Modified 0.5 mL centrifuge tubes had a central hole in the lid covered with black cloth for ventilation. Inside each tube, a bean leaf square infested with *Tetranychus urticae* eggs was placed on T‐shaped filter paper. To maintain leaf freshness, (c) 10 μL of filtered water was added daily to the filter paper. *P. persimilis* individuals (shown in orange) are not drawn to scale.
**Table S1.** Mean (±SEM) pre‐oviposition period (days), daily oviposition rate (eggs day^−1^) over a 10‐day observation period, and maximum daily oviposition rate (eggs day^−1^) of *Phytoseiulus persimilis* (F_4_) females in relation to their own ancestral dietary history, their partners' ancestral dietary history, and their partners' mating history.
**Table S2.** ART ANOVA results for the effects of F_4_ females' own ancestral dietary history (female‐line), partners' ancestral dietary history (male‐line), and partners' mating history (history) on pre‐oviposition period, daily oviposition rate, and maximum daily oviposition rate. Significant effects are indicated in bold (*P* < 0.05).
**Table S3.** ANOVA results for *Phytoseiulus persimilis* (F_5_) egg size in relation to paternal‐line ancestral dietary history, maternal‐line ancestral dietary history, and paternal mating history. Significant effects (*P* < 0.05) are shown in bold.
**Table S4.** ANOVA results for *Phytoseiulus persimilis* (F_5_) female developmental duration in relation to paternal‐line ancestral dietary history, maternal‐line ancestral dietary history, and paternal mating history. Significant effects (*P* < 0.05) are shown in bold.
**Table S5.** ANOVA results for *Phytoseiulus persimilis* (F_5_) female size at maturity (dorsal plate length) in relation to paternal‐line ancestral dietary history, maternal‐line ancestral dietary history, and paternal mating history. Significant effects (*P* < 0.05) are shown in bold.

## Data Availability

The data that supports the findings of this study are available in the supplementary material of this article.
