# Supplementary material for: Dietary restriction induces persistent multigenerational phenotypic effects in Phytoseiulus persimilis
Source: Pest Manag Sci. 2026 May 17;82(9):8725–33. doi: 10.1002/ps.70923 (PMC13453197; doi:10.1002/ps.70923)
Supplement: Supplementary file 1 [file PS-82-8725-s001.docx]

**Fig. S1.** Experimental rearing arenas for *Phytoseiulus persimilis*. (*a*) Rearing cells used for immature development and adult mating (1♀ + 1♂). Each cell comprised a plexiglass slide (25 × 20 × 1 mm) with a central cavity (6 mm diameter) covered by a smaller glass slide (20 × 15 × 1 mm). A black cotton cloth was affixed to the base for contrast and ventilation. Two metal clips held the slides together. (*b*) Rearing tubes used for individual adult maintenance. Modified 0.5 mL centrifuge tubes had a central hole in the lid covered with black cloth for ventilation. Inside each tube, a bean leaf square infested with *Tetranychus urticae* eggs was placed on T-shaped filter paper. To maintain leaf freshness, c. 10 μL of filtered water was added daily to the filter paper. *P. persimilis* individuals (shown in orange) are not drawn to scale.

Figure S1

**Appendices**

Table S1. Mean (± SEM.) pre-oviposition period (days), daily oviposition rate (eggs day⁻¹) over a 10-day observation period, and maximum daily oviposition rate (eggs day⁻¹) of Phytoseiulus persimilis (F₄) females in relation to their own ancestral dietary history, their partners’ ancestral dietary history, and their partners’ mating history.

| Male-line ancestral history | Female-line ancestral history | Male mating history | *n* | Pre-oviposition (days) | Daily oviposition rate (eggs/day) | Maximum daily oviposition (eggs/day) |
| --- | --- | --- | --- | --- | --- | --- |
| High | High | First | 10 | 1.8 ± 0.1 | 4.0 ± 0.2 | 5.0 ± 0.1 |
|  |  | Second | 8 | 1.3 ± 0.2 | 4.1 ± 0.1 | 5.0 ± 0.0 |
|  | Low | First | 9 | 1.7 ± 0.2 | 3.9 ± 0.1 | 5.1 ± 0.2 |
|  |  | Second | 10 | 1.3 ± 0.2 | 4.1 ± 0.1 | 5.3 ± 0.2 |
| Low | High | First | 9 | 1.6 ± 0.2 | 4.2 ± 0.1 | 5.1 ± 0.1 |
|  |  | Second | 9 | 1.6 ± 0.2 | 3.5 ± 0.2 | 4.9 ± 0.2 |
|  | Low | First | 10 | 1.5 ± 0.2 | 3.9 ± 0.1 | 5.2 ± 0.2 |
|  |  | Second | 9 | 1.6 ± 0.2 | 3.6 ± 0.3 | 5.1 ± 0.3 |

Table S2. ART ANOVA results for the effects of F₄ females’ own ancestral dietary history (female-line), partners’ ancestral dietary history (male-line), and partners’ mating history (history) on pre-oviposition period, daily oviposition rate, and maximum daily oviposition rate. Significant effects are indicated in bold (*p* < 0.05).

| Parameter | Pre-oviposition period | | | Daily oviposition | | | Maximum daily oviposition | | |
| --- | --- | --- | --- | --- | --- | --- | --- | --- | --- |
|  | *df* | *F* | *p* | *df* | *F* | *p* | *df* | *F* | *p* |
| Male-line | 1, 66 | 0.128 | 0.722 | 1, 66 | 2.792 | 0.099 | 1, 66 | 0.871 | 0.354 |
| Female-line | 1, 66 | 0.516 | 0.475 | 1, 66 | 0.166 | 0.685 | 1, 66 | 0.210 | 0.648 |
| History | 1, 66 | 1.756 | 0.190 | 1, 66 | 2.058 | 0.156 | 1, 66 | 0.013 | 0.911 |
| Male-line × Female-line | 1, 66 | 0.824 | 0.367 | 1, 66 | 0.075 | 0.785 | 1, 66 | 0.036 | 0.850 |
| Male-line × History | 1, 66 | 1.227 | 0.272 | 1, 66 | 4.676 | **0.034** | 1, 66 | 1.965 | 0.166 |
| Female-line × History | 1, 66 | 0.868 | 0.355 | 1, 66 | 2.176 | 0.145 | 1, 66 | 0.009 | 0.924 |
| Male-line × Female-line × History | 1, 66 | 1.257 | 0.266 | 1, 66 | 1.327 | 0.254 | 1, 66 | 0.001 | 0.979 |

Table S3. ANOVA results for *Phytoseiulus persimilis* (F₅) egg size in relation to paternal-line ancestral dietary history, maternal-line ancestral dietary history, and paternal mating history. Significant effects (*p* < 0.05) are shown in bold.

| Parameter | *df* | *F* | *p* |
| --- | --- | --- | --- |
| **Paternal-line** | 1, 232 | 9.088 | **0.003** |
| **Maternal-line** | 1, 232 | 28.228 | **< 0.001** |
| **History** | 1, 232 | 7.314 | **0.007** |
| Paternal-line × Maternal-line | 1, 232 | 2.096 | 0.149 |
| Paternal-line × History | 1, 232 | 1.164 | 0.282 |
| **Maternal-line × History** | 1, 232 | 34.986 | **< 0.001** |
| **Paternal-line × Maternal-line × History** | 1, 232 | 15.077 | **< 0.001** |

Table S4. ANOVA results for *Phytoseiulus persimilis* (F₅) female developmental duration in relation to paternal-line ancestral dietary history, maternal-line ancestral dietary history, and paternal mating history. Significant effects (*p* < 0.05) are shown in bold.

| Parameter | *df* | *F* | *p* |
| --- | --- | --- | --- |
| Paternal-line | 1, 71 | 3.153 | 0.080 |
| **Maternal-line** | 1, 71 | 5.654 | **0.020** |
| History | 1, 71 | 0.963 | 0.330 |
| Paternal-line × Maternal-line | 1, 71 | 1.595 | 0.211 |
| **Paternal-line × History** | 1, 71 | 8.995 | **0.004** |
| Maternal-line × History | 1, 71 | 0.134 | 0.715 |
| **Paternal-line × Maternal-line × History** | 1, 71 | 14.392 | **< 0.001** |

Table S5. ANOVA results for *Phytoseiulus persimilis* (F₅) female size at maturity (dorsal plate length) in relation to paternal-line ancestral dietary history, maternal-line ancestral dietary history, and paternal mating history. Significant effects (*p* < 0.05) are shown in bold.

| Parameter | *df* | *F* | *p* |
| --- | --- | --- | --- |
| Paternal-line | 1, 71 | 1.766 | 0.188 |
| **Maternal-line** | 1, 71 | 25.789 | **< 0.001** |
| History | 1, 71 | 0.720 | 0.399 |
| Paternal-line × Maternal-line | 1, 71 | 1.376 | 0.245 |
| Paternal-line × History | 1, 71 | 2.855 | 0.096 |
| Maternal-line × History | 1, 71 | 3.794 | 0.055 |
| **Paternal-line × Maternal-line × History** | 1, 71 | 8.803 | **0.004** |
